# Reflecting the Self: The Mirror Effect of Narcissistic Self-Regulation in Older Adults’ Evaluations of Empathic vs. Cold Socially Assistive Robots

**DOI:** 10.3390/bs16020164

**Published:** 2026-01-23

**Authors:** Avi Besser, Virgil Zeigler-Hill, Keren Mazuz

**Affiliations:** 1 Department of Communication Disorders, Jerusalem Multidisciplinary College, Jerusalem 91010, Israel; 2 Department of Psychology, Oakland University, Rochester, MI 48309, USA; zeiglerh@oakland.edu; 3 School of Management, Jerusalem Multidisciplinary College, Jerusalem 91010, Israel; kerenma@jmc.ac.il

**Keywords:** older adults, aging, socially assistive robots, human-robot interaction, perceived recognition, personality traits, narcissistic admiration, narcissistic rivalry, empathic behavior

## Abstract

Empathic behavior is increasingly incorporated into socially assistive robots, yet little is known about how older adults’ personality-based self-regulatory processes shape responses to such designs. The present study examined a recognition-based “mirror effect” framework of narcissistic self-regulation, referring to the ways individuals maintain a valued self-image through social feedback and acknowledgment. We focused on two core dimensions: narcissistic admiration, characterized by self-promotion and the pursuit of affirmation, and narcissistic rivalry, characterized by defensiveness, antagonism, and sensitivity to threat. Community-dwelling older adults (N = 527; *M*_age_ = 72.73) were randomly assigned to view a video of a socially assistive robot interacting in either an empathic or a cold manner. Participants reported their perceived recognition by the robot, defined as the subjective experience of feeling seen, acknowledged, and valued, as well as multiple robot evaluations (anthropomorphism, likability, perceived intelligence, safety, and intention to use). At the mean level, empathic robot behavior increased perceived recognition, anthropomorphism, and likability but did not improve perceived intelligence, safety, or intention to use. Conditional process analyses revealed that narcissistic admiration was positively associated with perceived recognition, which in turn predicted more favorable robot evaluations, regardless of robot behavior. In contrast, narcissistic rivalry showed a behavior-dependent pattern: rivalry was associated with reduced perceived recognition and less favorable evaluations primarily in the empathic condition, whereas this association reversed in the cold condition. Importantly, once perceived recognition and narcissistic traits were accounted for, the cold robot was evaluated as more intelligent, safer, and more desirable to use than the empathic robot. Studying these processes in older adults is theoretically and practically significant, as later life is marked by shifts in social roles, autonomy concerns, and sensitivity to interpersonal evaluation, which may alter how empathic technologies are experienced. Together, the findings identify perceived recognition as a central psychological mechanism linking personality and robot design and suggest that greater robotic empathy is not universally beneficial, particularly for users high in rivalry-related threat sensitivity.

## 1. Introduction

Over the past two decades, human–robot interaction (HRI) research has expanded from a primary focus on task efficiency to a broader examination of the social, affective, and relational dimensions of how humans respond to robots (i.e., socially assistive robots; SAR). Early studies within the “computers as social actors” paradigm demonstrated that people readily apply human social norms to machines, showing politeness, reciprocity, and other social responses even when interacting with minimal social cues (Reeves & Nass, 1996). Subsequent work extended these findings to embodied agents and social robots, suggesting that embodiment and behavioral cues further amplify social attributions and relational expectations (Breazeal, 2003; Fong et al., 2003; Mazuz & Yamazaki, 2024). As robots become increasingly integrated into everyday life, serving as companions or assistants to caregivers, their success depends not only on technical competence but also on how users perceive their social presence and emotional attunement (see Berrezueta-Guzman et al., 2025; Park & Whang, 2022). As emphasized by Fuchs (2024), the rise in empathic artificial agents can be understood as a form of “technological narcissism,” in which machines function as mirrors reflecting users’ needs for validation and emotional recognition, situating empathic HRI within a broader sociotechnical context of self-referentiality and emotional outsourcing (Fuchs, 2024).

Focusing on older adults is especially relevant in this context. The global population is rapidly aging, with life expectancy rising in almost every region as indicated by the World Health Organization (WHO, 2025b) and the United Nations (UN, 2022). By 2050, one in six people worldwide will be over age 65, heightening the urgency of social and healthcare transformations (UN, 2023; WHO, 2025a). Socially assistive robots have emerged as promising tools to support autonomy, emotional well-being, and social connectedness among older individuals, especially those facing challenges such as isolation, frailty, or cognitive decline (Broadbent, 2017; Papadopoulos et al., 2020). Narrative reviews of social telepresence and communication technologies similarly suggest that robot-mediated contact can help mitigate loneliness and social isolation in older adults (Isabet et al., 2021). Beyond practical assistance, empathic robotic design may help fulfill older adults’ fundamental need for belonging and recognition (Baumeister & Leary, 1995). Understanding how older adults with varying personality profiles, such as levels of narcissism, respond to empathic versus cold robots is therefore critical for designing technologies aligned with users’ emotional and psychological needs. Despite the rapid growth of this literature, little is known about how narcissistic personality traits shape older adults’ responses to empathic versus cold robots, or whether these responses are driven by their subjective sense of being recognized by the robot.

Recent scholarship has focused on empathy as a core design principle, particularly in assistive and healthcare robotics (Papadopoulos et al., 2020). Empathic cues such as adaptive feedback, eye contact, tone modulation, and emotional responsiveness typically increase perceived warmth and responsiveness, which may, under some conditions, translate into a deeper experience of being acknowledged and valued. Conversely, robots that act impersonally often evoke distrust or discomfort (Park & Whang, 2022). Yet, as this research suggests, individual differences in personality significantly influence how people interpret and respond to such social cues. Some users readily attribute warmth and agency to artificial agents, whereas others remain detached or skeptical. Personality traits thus moderate trust, acceptance, and engagement with robots (de Graaf & Ben Allouch, 2013; Henschel et al., 2021; Rossi et al., 2020).

Within this expanding literature, one particularly relevant but under-examined trait is narcissism. Narcissism, defined by grandiosity and vulnerability to ego threat, reflects a chronic need for validation and admiration (Back et al., 2013; Morf & Rhodewalt, 2001). Because social robots are designed to convey attention, empathy, and recognition, narcissism provides a rich theoretical lens for understanding divergent user reactions.

The present study therefore investigates how narcissistic admiration and rivalry shape older adults’ responses to empathic versus cold robot behavior and whether these effects are carried by their perceived recognition, that is, the extent to which they feel seen, acknowledged, and respected by the robot.

### 1.1. Literature Review

#### 1.1.1. Empathy and Social Cues in Human–Robot Interaction

Empathy in HRI refers to a robot’s ability to perceive and respond appropriately to a user’s emotional state, thoughts, and situation, and to generate affective or cognitive responses that elicit positive perceptions (Park & Whang, 2022). This often involves multimodal social cues such as contingent verbal responses, prosodic modulation, gaze, and posture that convey attentiveness and concern (Broadbent, 2017; Stock-Homburg, 2021). Studies in healthcare, domestic, and educational contexts suggest that robots displaying such empathic cues are evaluated as more likable and socially engaging than neutral counterparts, and are more likely to be accepted and used over time (Henschel et al., 2021; Papadopoulos et al., 2020; Rossi et al., 2020). Complementing this work, recent studies with assistive and companion robots in older adult care settings indicate that such systems can be both technically feasible and acceptable to many older users (Bevilacqua et al., 2021; Łukasik et al., 2021). Perugia et al. (2020) further show that individual differences in empathy and openness shape emotional contagion and rapport during HRI, underscoring that empathic design must be understood in interaction with user characteristics (Perugia et al., 2020).

Stock-Homburg’s (2021) review of 70 empirical studies demonstrated that emotional expressivity, facial movement, gaze, and vocal tone systematically enhance perceived intelligence and warmth (Stock-Homburg, 2021). Robots capable of affective attunement are judged more socially competent, whereas those lacking emotional display seem mechanical or alienating. These findings align with social presence theory (Short et al., 1976), emphasizing that responsiveness and emotional congruence underpin human connection to artificial agents.

To reduce conceptual overlap, we distinguish between (a) empathic behavior cues as the experimentally manipulated interactional style (e.g., prosody, responsiveness, gaze), (b) warmth and social presence as broader evaluative impressions of the robot’s socio-emotional orientation, and (c) perceived recognition as the focal self-referential appraisal examined in the present study—namely, the subjective experience of feeling seen, acknowledged, and valued by the robot. Conceptually, perceived recognition is therefore not treated as synonymous with warmth. Rather, it reflects a deeper relational judgment that may or may not follow from empathic cues, depending on users’ motivational orientations and defensive self-regulatory processes. Empathy is especially consequential in assistive settings. Papadopoulos et al. (2020) identified emotional engagement and personalization as key factors facilitating robot acceptance among older adults and patients. Perceived caring or attentiveness reduced loneliness and increased willingness to engage. Participatory studies with older adults further indicate that acceptance of companion and assistive robots depends strongly on how well these technologies fit users’ emotional needs and everyday routines (Zguda et al., 2025). These contemporary findings resonate with Baumeister and Leary’s (1995) “need to belong” hypothesis, suggesting that empathic design satisfies a fundamental human drive for interpersonal acknowledgment, even when the source is artificial (Baumeister & Leary, 1995).

Building on this motivational account, recognition can be understood not only as a behavioral affordance of empathic design but also as a deeper relational and symbolic process. Beyond its operational role in HRI, the concept of recognition also holds rich theoretical significance in social philosophy. Scholars like Honneth (1996) and Fraser (2000) have emphasized recognition as a fundamental human need linked to identity formation, social justice, and dignity. Integrating this lens may deepen our understanding of how empathic robots not only simulate interpersonal care but also potentially engage with core elements of personal and social identity (Fraser, 2000; Honneth, 1996).

Hsieh and Cross (2022) add that users with higher trait empathy experience stronger emotional contagion during HRI, which suggests that empathic robot design may be particularly effective for such individuals (Hsieh & Cross, 2022). By analogy, this sensitivity to empathic cues may be especially relevant for socially sensitive populations such as older adults, for whom recognition and relational attunement may play a central role in technology acceptance (Hsieh & Cross, 2022).

At the same time, recognition in HRI is not uniformly experienced as positive. Some scholars have noted that overly empathic robotic responses may also provoke discomfort or feelings of inauthenticity, particularly when users perceive the empathy as simulated or instrumental (Fuchs, 2024). This tension highlights the dual-edged nature of social robotics: they can affirm human needs, but also expose the limits of artificial care.

To address this complexity, we distinguish between (a) *empathic design cues*, observable signals such as tone, gaze, and supportive language that convey warmth or responsiveness; (b) *perceived recognition*, a subjective experience of being seen, acknowledged, and respected; and (c) *evaluative outcomes*, such as anthropomorphism, likability, perceived intelligence, safety, and intention to use. Empathic cues can shape impressions of warmth or social presence, but recognition captures a deeper self-relevant appraisal (i.e., “this agent acknowledges me as a person”), which is conceptually separable from simply finding the robot pleasant or humanlike.

From this perspective, an older adult’s perceived recognition, the extent to which they feel seen, heard, and acknowledged by the robot, can be considered a central psychological mechanism through which empathic HRI fosters positive evaluations and sustained engagement.

#### 1.1.2. Personality and Individual Differences in HRI

Personality traits play a central role in shaping how individuals evaluate and engage with robots. Previous work in HRI demonstrated that user characteristics and prior attitudes substantially influence the acceptance of social robots in everyday contexts (de Graaf & Ben Allouch, 2013). Subsequent empirical and meta-analytic research has shown that broad personality dimensions such as extraversion, agreeableness, and openness are positively associated with robot acceptance and trust, whereas neuroticism is often linked to more skeptical or avoidant responses (Esterwood et al., 2021; Joshi et al., 2023; Kabacińska et al., 2025). For example, Zhang et al. (2020) found that people attribute greater warmth and sociability to social robots than to industrial robots, and that these perceptions vary systematically as a function of individual differences (Zhang et al., 2020).

Theoretical models of HRI further suggest that personality shapes not only overt evaluations of social robots but also underlying emotional and motivational engagement with these artificial agents (Henschel et al., 2021). Dispositional tendencies related to curiosity, cooperativeness, and perspective-taking are associated with stronger emotional resonance, increased anthropomorphism, and greater perceptions of intentionality, whereas tendencies toward emotional withdrawal or low empathy are linked to more detached or instrumental responses (e.g., Jones, 2021; Perugia et al., 2020). In this way, personality functions as a boundary condition shaping social cognition and affective responses toward nonhuman agents.

Consistent with this perspective, research on individual differences in HRI and attitudes toward Artificial Intelligence (AI) has commonly relied on broad trait frameworks to characterize personality differences (e.g., Big 5 personality dimensions; Esterwood et al., 2021; Zhang et al., 2020). Across studies, tendencies reflecting sociability, agreeableness, emotional stability, and openness are associated with more positive orientations toward social robots and other embodied AI agents used in everyday contexts, whereas heightened emotional distress is more often linked to skepticism or avoidance in human–robot interaction settings (Esterwood et al., 2021; Joshi et al., 2023; Kabacińska et al., 2025). In the present study, we focus specifically on socially assistive robots as an embodied class of AI, because attitudes toward “AI” can differ meaningfully from responses to physically embodied agents that display relational cues.

#### 1.1.3. Narcissism and Interpersonal Dynamics

Narcissism represents a paradoxical blend of self-enhancement and fragility. Morf and Rhodewalt’s (2001) dynamic model describes narcissistic individuals as reliant on external validation to maintain inflated self-views (Morf & Rhodewalt, 2001). Back et al. (2013) expanded this idea through the Narcissistic Admiration and Rivalry Concept (NARC), distinguishing between assertive self-promotion (narcissistic admiration) and defensive self-protection (narcissistic rivalry). Narcissistic admiration predicts charm and confidence, whereas narcissistic rivalry predicts hostility and rejection sensitivity (Back et al., 2013).

Empirical studies validate this duality. Neuroscientific work suggests that narcissistic traits are linked to heightened neural sensitivity to self-relevant feedback: individuals high in narcissism show stronger neural responses to social exclusion and to self-referential processing (Cascio et al., 2015; Jauk et al., 2017). At the behavioral level, Gnambs and Appel (2018) reported that narcissism is associated with intensified reactions to social feedback in online communication, such as stronger affective responses to likes and comments (Gnambs & Appel, 2018). However, recent work by Armbruster et al. (2025) indicates that evaluations of human versus artificial moral agents are driven primarily by agreement with the agent’s choice and general attitudes toward robots, rather than by specific traits such as narcissism (Armbruster et al., 2025). This pattern suggests that contextual cues and perceived recognition may be particularly important when people judge artificial agents.

#### 1.1.4. Integrating Narcissism with Empathic vs. Cold Robot Behavior

When applied to HRI, narcissism may predict distinct reactions to empathic versus cold robotic behavior. Empathic robots usually provide recognition cues such as eye contact, adaptive tone, and responsive feedback that affirm user visibility and importance. This research assumes that for narcissistic users, these cues engage admiration pathways, producing satisfaction and attraction. In contrast, cold, unresponsive behavior may evoke ego threat and rivalry-based defensiveness.

Recent work on intelligent agents and socially strategic robots further highlights the capacity of artificial systems to engage in morally and relationally consequential exchanges. For example, the “Nice and Nasty Theory of Mind” framework (D’Angelo et al., 2023) describes how robots can be designed to exhibit cooperative versus disruptive social behaviors. Empirical findings within this framework indicate that users respond with increased suspicion and emotional distancing when robots violate expectations of prosociality (e.g., D’Angelo et al., 2023). Collectively, this work highlights that the social framing of robotics, supportive or self-serving, plays a critical role in shaping attributes of mental states and intentions, above and beyond its purely functional capabilities.

Similarly, Sgorbissa et al. (2024) discussed “Machiavellian robots” that can simulate manipulative, self-serving social cues, illustrating how anthropomorphic AI may mirror human tendencies toward strategic self-presentation (Sgorbissa et al., 2024). Xiao et al. (2025) argued that narcissistic tendencies can, under some conditions, enhance perceived compatibility with AI partners, as responsive systems are experienced as “charming” collaborators (Xiao et al., 2025). At the same time, Poushneh et al. (2024) found that empathic voice-AI responses were particularly effective for users higher in narcissism, whereas cold or dismissive agents could trigger heightened defensiveness (Poushneh et al., 2024). Together, these studies suggest that artificial agents can both reinforce and challenge self-enhancement motives, depending on whether they are perceived as recognizing and affirming the user.

Crucially, this research hypothesis is that these processes are likely to unfold through users’ perceived recognition: empathic robots should enhance the sense of being acknowledged, thereby amplifying the positive, admiration-based aspects of narcissism, whereas cold robots should undermine perceived recognition, potentially activating rivalry-based responses such as suspicion, devaluation, and emotional distancing. Taken together, these considerations lead us to propose a moderated-mediation “mirror effect” model in which older adults’ narcissistic admiration and rivalry predict their evaluations of a SAR indirectly through perceived recognition, and in which the robot’s behavioral style (empathic vs. cold) moderates this indirect pathway. Yet these dynamics also invite broader ethical and psychological questions. To what extent might empathic SAR, designed to provide recognition, inadvertently reinforce dependency or externalize self-worth? This study opens the door to further exploration of the potential risks associated with relying on SAR as sources of emotional validation and self-affirmation. We return to these ethical implications in the Discussion, where we consider design safeguards and the potential risks of “imposed empathy” for users who may experience relational cues as evaluative or autonomy-threatening.

We assume that empathic behavior is expected to strengthen the positive admiration–recognition–evaluation pathway, whereas cold behavior is expected to strengthen the negative rivalry–recognition–evaluation pathway. Specifically, we expect narcissistic admiration to show its most positive associations with robot evaluation when empathic behavior enhances perceived recognition, whereas narcissistic rivalry should show its most negative associations with robot evaluation when cold behavior reduces perceived recognition. This “mirror effect” positions empathy in HRI as a symbolic mechanism reflecting the dynamic interplay between self-importance and perceived recognition, thereby linking narcissistic self-regulation to empathic design.

### 1.2. The Present Study

Building on the above framework, the present study examines how narcissistic admiration and rivalry shape older adults’ reactions to a SAR that behaves either empathically or coldly. In the present design, the experimental manipulation targets the robot’s behavioral style (empathic vs. cold), whereas perceived recognition is measured as the user’s subjective psychological response through which these style cues may translate into downstream evaluations. Accordingly, perceived recognition is treated as a mechanism (mediator) rather than as a manipulation check. We focus on perceived recognition (i.e., feeling seen, understood, and respected by the robot) as a key psychological mechanism through which robot behavior influences user evaluations.

**Figure 1:**
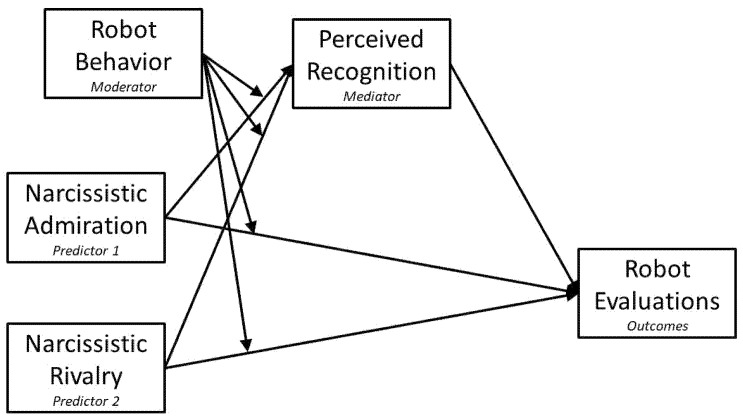
Proposed moderated mediation (“mirror effect”) model. Narcissistic admiration and rivalry (X) predict perceived recognition (M), which in turn predicts robot evaluation (Y; anthropomorphism, likability, perceived intelligence, safety, and intention to use). Robot behavioral style (empathic vs. cold; W) moderates the X → M path, yielding conditional indirect effects on Y.

Specifically, we propose that older adults’ narcissistic admiration and rivalry will predict how strongly they feel recognized by the robot, and that this perceived recognition will, in turn, shape their evaluations of the robot. The robot’s behavioral style (empathic vs. cold) is expected to moderate the links between narcissistic traits and perceived recognition, thereby producing conditionally indirect associations (“mirror effect”) between narcissism and robot evaluation (see Figure 1 for the conceptual model).

Based on the theoretical and empirical literature reviewed above, we derived the following hypotheses.

#### Hypotheses

The proposed relationships were tested using PROCESS Model 8 (Hayes, 2022), which examines moderated mediation effects by estimating indirect paths conditional on the moderator (robot behavior).

**H1.**  *Older adults higher in narcissistic admiration will evaluate a SAR more positively via perceived recognition. Specifically, narcissistic admiration will be positively associated with perceived recognition, and perceived recognition will predict more favorable evaluations of the robot, consistent with an indirect (mediated) association via perceived recognition.*

**H2.**  *The association between older adults’ narcissistic admiration and perceived recognition will be moderated by the robot’s behavioral style. Specifically, narcissistic admiration will be more strongly associated with perceived recognition when the robot behaves in an empathic manner than when it behaves in a cold manner.*

**H3.**  *Older adults higher in narcissistic rivalry will evaluate a SAR less favorably via diminished perceived recognition. Specifically, narcissistic rivalry will be negatively associated with perceived recognition, and perceived recognition will predict less favorable evaluations of the robot, consistent with an indirect (mediated) association via diminished perceived recognition.*

**H4.**  *The association between older adults’ narcissistic rivalry and perceived recognition will be moderated by the robot’s behavioral style. Specifically, narcissistic rivalry will be more strongly associated with diminished perceived recognition when the robot behaves in a cold manner than when it behaves in an empathic manner.*

### 1.3. Theoretical Model: The Mirror Effect

The proposed moderated mediation model posits that older adults’ narcissistic admiration and rivalry predict their evaluations of a SAR indirectly through their perceived recognition by the robot. Higher perceived recognition is expected to predict more favorable evaluations of the robot, whereas lower perceived recognition is expected to predict less favorable evaluations (H1, H3). The robot’s behavioral style (empathic vs. cold) is conceptualized as a moderator of the pathway from narcissistic traits to perceived recognition, such that the resulting indirect association with robot evaluations is conditional on the robot’s behavior. Specifically, empathic behavior is expected to strengthen the positive indirect association between narcissistic admiration and favorable robot evaluations via heightened perceived recognition, whereas cold behavior is expected to strengthen the negative indirect association between narcissistic rivalry and unfavorable robot evaluations via diminished perceived recognition (H2, H4). This mirror-effect framework integrates empathic design and narcissistic self-regulation by proposing that the same narcissistic tendencies can lead to either enhanced engagement or defensive rejection, depending on whether the robot’s behavior conveys recognition or indifference.

Grounded in Honneth’s (1996) recognition theory (Honneth, 1996) and Fraser’s (2000) social-justice perspective (Fraser, 2000), the model conceptualizes perceived recognition as a fundamental psychological need rather than a superficial interaction cue, thereby highlighting the need for adaptive social design that takes into account individual differences in self-focused motivation.

## 2. Materials and Methods

### 2.1. Participants

The sample consisted of 637 community-dwelling older adults aged 65 years and above. To ensure a balanced gender distribution, the sample included 316 men and 321 women. Participants were recruited through multiple channels, including senior community centers, online forums for retirees, mailing lists associated with adult-education programs, and “iPanel,” a well-established local online panel in which individuals voluntarily register to complete surveys in exchange for monetary compensation.

Eligibility criteria required participants to have normal or corrected-to-normal vision and hearing and no diagnosed cognitive impairment. Participation was entirely voluntary. Participants were provided 10 ILS (approximately 2.5 USD) for participating.

All data were collected through a secure online platform. After providing informed consent, participants completed a brief set of questionnaires, watched a video stimulus, and then completed some additional questionnaires. They were informed that participation was voluntary, that they could discontinue at any point, and that no personally identifiable data would be recorded.

Participant exclusions for inattentive or invalid responding were conducted using pre-specified criteria. Thirty-six participants were removed for exhibiting univariate outlier values on one or more variables. An additional 39 participants were excluded due to highly inconsistent response patterns, as indicated by unusually large inter-item standard deviations. Thirty-five participants demonstrated evidence of ‘straightlining’ (i.e., providing the same response across a large number of items), identified through longstring analysis, and were removed accordingly.

After applying these exclusion criteria, the final analytic sample consisted of 527 older adults (271 men, 256 women), who were randomly assigned to view either the cold robot condition (*n* = 257; 131 men, 126 women) or the empathic robot condition (*n* = 270; 140 men, 130 women). Importantly, participants were not selected based on narcissism, and no exclusions were made based on narcissism levels. The measure of narcissism was administered to the full initial sample to capture natural, continuous variability in narcissistic admiration and rivalry, consistent with an individual-differences (rather than diagnostic) approach. Preliminary analyses that retained all excluded participants yielded results that were highly consistent with those reported for the final sample.

Participants ranged in age from 66 to 86 years (*M* = 72.73, *SD* = 4.69). On average, they reported having 2.90 children, relatively low levels of loneliness (*M* = 22.58 on a 0–100 scale), and relatively high perceived social support (*M* = 73.41 on a 0–100 scale). With respect to prior exposure to robotics, 41.2% reported previous experience with robots, whereas 58.8% reported no prior experience.

Educational attainment was generally high: 11.0% reported less than a high school education, 27.7% completed high school, 36.4% held a bachelor’s degree, 21.6% held a master’s degree, and 3.2% held a doctoral degree or equivalent. Regarding employment status, most participants reported being homemakers (64.1%), followed by full-time employment (17.8%) and part-time employment (14.0%). Smaller proportions reported being unemployed (1.6%), enrolled in school (0.6%), or retired (1.9%).

Most participants were married (71.7%), with others reporting being divorced (13.9%), widowed (7.8%), single (2.8%), cohabiting (2.5%), separated (0.6%), or dating (0.8%). Household income was broadly distributed, with 12.9% reporting very high income, 25.0% somewhat high, 32.3% moderate, 18.0% somewhat low, and 11.8% very low income. Finally, the majority of participants identified as secular (68.1%), followed by traditional (20.1%), religious (8.2%), and ultra-orthodox (3.6%).

A priori power analysis (G*Power 3.1) indicated that a minimum of 395 participants would be sufficient to detect a small moderated-mediation effect (f^2^ = 0.02, α = 0.05, 1 − β = 0.80). The final sample (N = 527) therefore provided excellent statistical power (>0.99) for all planned analyses.

### 2.2. Materials

#### 2.2.1. Video Stimuli

**Figure 2:**
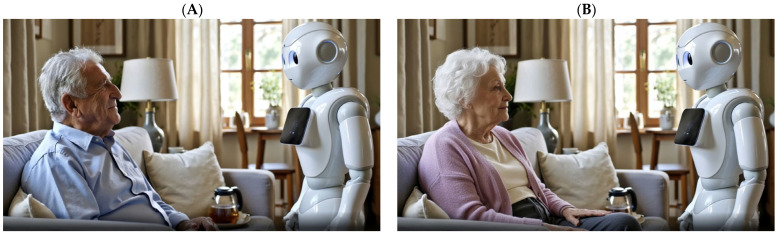
Experimental stimuli depicting older adults—Mr. Cohen (**A**) and Ms. Cohen (**B**)—interacting with the humanoid service robot Novi in a home-like living-room setting.

Two brief video clips (each approximately 1 min and 12 s long) served as the experimental stimuli. Both depicted an identical scenario in which an older adult interacted with a SAR named Novi while requesting assistance in preparing tea (Mr. Cohen for male participants; Ms. Cohen for female participants; See Figure 2).

We selected a tea-preparation scenario because it is a familiar, culturally recognizable activity that naturally involves both instrumental assistance and interpersonal sensitivity, making it well suited for studying HRI among older adults. Using a simple, low-stakes task helped keep attention on the manipulated interaction style (empathic vs. cold) rather than on task difficulty or performance demands.

The only difference between the two videos was the robot’s emotional display:

1. Empathic Condition (*n* = 270; 140 men and 130 women). In this version, Novi spoke in a warm and gentle tone, maintained eye contact, used natural prosodic modulation, and offered supportive verbal cues (e.g., “I can see your hands are trembling a little, take your time, I’m right here with you.”).
2. Cold Condition (*n* = 257; 131 men and 126 women). In this version, Novi spoke in a flat, mechanical tone, avoided eye contact, minimized verbal cues, and omitted any empathic references (e.g., “Acknowledged. Preparing tea. Please specify type.”).

The two clips were identical in duration, setting, camera angle, dialogue structure, and visual design, differing only in the robot’s emotional tone and accompanying social cues. The older adult in the video was gender-matched to each participant to facilitate basic situational identification with the scenario (i.e., viewing an older adult of the same gender), and to avoid introducing an extraneous source of distraction unrelated to the robot’s behavior. This design choice was intended to support immersion in the scenario rather than to test gender-related effects.

#### 2.2.2. Final Stimulus Set and Standardization

The AI tools listed below were used during stimulus development to generate multiple candidate renderings of the same scripted interaction. Importantly, participants were not exposed to tool-specific versions. Instead, we selected and exported a single final clip per experimental condition (empathic vs. cold) for each protagonist gender (Mr. Cohen/Ms. Cohen), resulting in four finalized videos (2 conditions × 2 protagonist genders). Each participant viewed one full-screen clip only, matched to their gender, and thus the experimental manipulation was solely the robot’s behavioral style. Although vignette-based stimuli cannot reproduce real-time reciprocity, this approach enabled rigorous standardization of social cues (dialogue, timing, and expressive behavior) that is difficult to achieve reliably with a physical robot, thereby isolating behavioral style as the key experimental manipulation.

#### 2.2.3. AI Video-Generation Tools and Production Pipeline

To create photorealistic stimuli while holding content constant, we used eight AI video platforms: Veo 3.1 (Google DeepMind, London, UK), Kling AI (Kuaishou Technology, Beijing, China), Sora 2 (OpenAI, San Francisco, CA, USA), Pictory (Bothell, WA, USA), InVideo AI (InVideo, San Francisco, CA, USA), Runway Gen-3 (Runway AI, New York, NY, USA), HeyGen (HeyGen Technology Inc., Los Angeles, CA, USA), and Hailuo AI (MiniMax, MiniMax, Shanghai, China). We relied on multiple platforms because no single generator consistently produced high-quality, constraint-satisfying outputs across all required elements (stable characters, lip-sync, gaze behavior, and scene continuity) while preserving an identical script and environment. Using several tools allowed us to generate multiple candidate renderings of the same scripted interaction and then select the best-matched final clips for experimental use. These platforms are cloud-based text-to-video or script-to-avatar systems that synthesize short photorealistic clips from textual prompts and reference images. For each tool, we applied the same prompts, storyboard, reference frame, dialogue script, target video length (~60 s), aspect ratio (16:9), and resolution (1080p). We further constrained avatars’ appearance, camera framing, and background environment to be as similar as technically possible, such that the scenario parameters (avatars, dialogue, timing, and scene structure) were effectively held constant across generators and only model-specific rendering differences varied. Importantly, the AI platforms were used during stimulus development; participants were not exposed to tool-specific versions. Instead, the finalized videos (empathic vs. cold; gender-matched protagonist) were used consistently across participants within each condition, with the protagonist gender-matched to the participant. Apart from grammatical gender marking and the protagonist’s name (Mr. Cohen vs. Ms. Cohen), the scripts were identical in wording, structure, timing, and content across versions.

Following generation, the clips underwent post-processing. Specifically, segments in which the robot was speaking were slowed to 0.85× speed. The same time-stretch procedure was applied identically to both conditions, using pitch-preserving processing, to avoid condition-specific changes in perceived vocal tone. This adjustment was made to enhance the comprehensibility of the voice for an elderly audience and to allow on-screen captions to be displayed for a longer duration, yielding a final video duration of approximately 1 min and 12 s.

##### Manipulation Validation (Expert Ratings)

To minimize demand characteristics and potential priming, we did not administer a direct manipulation-check item to participants (e.g., “How empathic was the robot?”). Because the focal outcomes included perceived recognition (i.e., feeling personally noticed, acknowledged, and valued by the robot) and downstream evaluations of the robot, explicitly prompting participants to evaluate the robot’s empathy could have increased salience of the intended contrast between conditions and influenced subsequent ratings. Consistent with our conceptualization of perceived recognition as a psychological mechanism (mediator) rather than a manipulation check, we therefore validated the manipulation using independent expert ratings.

Six expert judges (three women and three men), all professionals from therapeutic fields (e.g., clinical psychology, social work), independently evaluated the stimulus videos. Each judge viewed two clips, one from the empathic behavioral style condition and one from the cold behavioral style condition, while viewing only the gender-matched version of the older adult protagonist (i.e., Mr. Cohen clips were rated by male judges; Ms. Cohen clips were rated by female judges), mirroring the gender-matched presentation used in the experiment. Judges rated the robot’s expressed empathy on a 0–100 scale (0 = not at all empathic, 100 = very empathic). Ratings were consistently higher for the empathic behavioral style (*M* = 88.33, *SD* = 2.07) than for the cold behavioral style (*M* = 47.50, *SD* = 5.24). Inter-rater reliability was excellent (ICC(2,k) = 0.996, two-way random effects, absolute agreement), indicating very high agreement among judges. A paired-samples *t*-test indicated that this difference was statistically significant, t(5) = 15.12, *p* < 0.001, with a very large effect size (Cohen’s dz = 6.17), supporting the intended manipulation of the robot’s behavioral style.

These expert ratings establish the intended contrast in the stimulus properties (expressed empathy) but do not capture participants’ subjective perception of the interaction in situ. Accordingly, we treat the judge-based validation as evidence of manipulation integrity rather than as a participant-level manipulation check. Future studies can strengthen internal validity by adding a brief, unobtrusive end-of-survey assessment of perceived warmth/attunement (e.g., 2–3 items) and/or comprehension-based checks that do not overlap conceptually with perceived recognition.

#### 2.2.4. Pre-Manipulation Questionnaires

All measures were administered in Hebrew and were translated from the original English versions using a standard translation–back-translation procedure to ensure semantic and conceptual equivalence.

##### Demographic Questionnaire

Participants completed a comprehensive demographic questionnaire that assessed age, gender, education level, religiosity, monthly income, occupational status, marital status, number of children, and prior experience with digital or robotic technologies. In addition, participants completed two single items assessing their sense of loneliness and the perceived availability of social support, each rated on a scale ranging from 0% (not at all) to 100% (very much). These indicators allowed for detailed characterization of the sample and potential control variables in the analyses.

##### Narcissistic Admiration and Rivalry

Narcissism was assessed using the Narcissistic Admiration and Rivalry Questionnaire (NARQ; Back et al., 2013). The NARQ is a self-report instrument designed to capture two central dimensions of narcissism: narcissistic admiration (9 items; e.g., “I am great”) and narcissistic rivalry (9 items; e.g., “I want my rivals to fail”). Participants rated their agreement with each statement on a 6-point Likert scale ranging from 1 (not agree at all) to 6 (agree completely). Subscale scores were computed as the mean of the relevant items, with higher scores indicating greater levels of narcissistic admiration or rivalry. The NARQ has demonstrated strong internal consistency and robust construct validity across diverse samples (e.g., Back et al., 2013). In the present study, internal consistency (Cronbach’s alpha) was α = 0.83 for narcissistic admiration and α = 0.82 for narcissistic rivalry.

#### 2.2.5. Post-Manipulation Questionnaires

##### Perceived Recognition Scale

In this context, perceived recognition refers to feeling personally noticed, acknowledged, and valued by the robot during the interaction. A new 6-item Perceived Recognition Scale (PRS) was developed specifically for this study. Item content was grounded in recognition theory (Honneth, 1996) and subsequently evaluated for content validity by two experts in social robotics. The scale assessed participants’ subjective sense of being seen, acknowledged, and appreciated by the robot (e.g., “The robot seemed to notice my emotional state,” “The robot seemed to value me as an individual”). This scale was designed to capture recognition as self-relevant acknowledgment (being “seen” and respected) rather than general warmth, likability, or anthropomorphic impressions. Items were rated on a 7-point scale ranging from 1 (strongly disagree) to 7 (strongly agree), and higher mean scores reflected stronger perceived recognition. An exploratory factor analysis using principal axis factoring with direct oblimin rotation indicated a clear single-factor structure (eigenvalue = 4.32), accounting for 72.02% of the total variance. The scale demonstrated excellent internal consistency in the present sample (α = 0.92). Conceptually, the PRS captures a self-relevant experience of acknowledgment (being noticed, seen, and valued) and is treated as distinct from downstream evaluative impressions (e.g., anthropomorphism or likability). Conceptually, this reflects a self-referential appraisal of being seen, acknowledged, and respected (Fraser, 2000; Honneth, 1996) and is therefore distinct from surface-level warmth or general likability; the PRS was developed to capture this deeper recognition experience as the proximal mechanism in the proposed model. The full item set and factor loadings are reported in Appendix A.

##### Robot Interaction Evaluation

Perceptions of the robot were assessed using the Robot Interaction Evaluation Scale (RIES) an adapted version of the Godspeed Series (Bartneck et al., 2008). The scale consists of 25 items measuring five key dimensions: anthropomorphism (5 items; e.g., “Fake” to “Natural”), likability (5 items; e.g., “Dislike” to “Like”), perceived intelligence (5 items; e.g., “Incompetent” to “Competent”), safety (5 items; e.g., “Anxious” to “Relaxed”), and intention to use (5 items; e.g., “I would avoid using this robot” to “I would use this robot”). Participants rated each item on 7-point semantic-differential scales. Subscale scores were computed as the mean of the corresponding items, with higher scores indicating more positive evaluations of the robot. The Godspeed scales have shown excellent internal consistency in previous research, with subscale reliabilities typically exceeding α = 0.85, and they have been widely used to assess HRI experiences. In the present study, internal consistency was α = 0.86 for anthropomorphism, α = 0.90 for likability, α = 0.92 for perceived intelligence, α = 0.91 for safety, and α = 0.97 for intention to use.

### 2.3. Measurement Validity for an Older Adult Population

To ensure that the instruments used in this study were appropriate for an older adult sample (aged 65+), we reviewed prior literature regarding their psychometric application in aging and HRI contexts. The NARQ has been included in a large-scale study with over 250,000 participants aged 13 to 77 (Weidmann et al., 2023), which confirmed the stability of its narcissistic admiration and narcissistic rivalry dimensions into later adulthood, thereby supporting its use with older adult samples. In addition, the Godspeed scales have been extensively used to assess anthropomorphism, animacy, likability, perceived intelligence, and perceived safety in HRI research, including work involving older individuals (e.g., Broadbent, 2017).

### 2.4. Procedure

Participants completed the study individually via a secure online session. After reading and providing online informed consent, they completed the Demographic Questionnaire, the NARQ, and a measure of basic personality dimensions (i.e., mini-IPIP scales; Donnellan et al., 2006) that was not relevant to the present analyses. To avoid order effects, the presentation of questionnaires was counterbalanced across participants.

Participants were then randomly assigned to one of two experimental video conditions: empathic robot (*n* = 270; 140 men and 130 women) or cold robot (*n* = 257; 131 men and 126 women). Each participant watched one video clip only, presented full-screen on their personal computer.

Before viewing the clip, participants received the following instruction:

*“Please watch the following interaction carefully and imagine that you are in Mr. Cohen’s [Ms. Cohen’s] place. Afterward, you will answer several questions about how you felt during the interaction.”*

Immediately after viewing the clip, participants completed the Perceived Recognition Scale and the Robot Interaction Evaluation Scale.

### 2.5. Design

A between-subjects experimental design was used. The primary manipulated independent variable was robot behavior (empathic vs. cold). Narcissistic admiration and narcissistic rivalry served as the focal predictors with perceived recognition as the mediator and robot behavior functioning as the moderator of the indirect pathway. The hypothesized model corresponds to a moderated mediation framework.

### 2.6. Ethical Considerations

All procedures complied with the Declaration of Helsinki and institutional ethical guidelines (IRB) for psychological research with human participants. Participation was voluntary, with the right to withdraw at any time without penalty. No personal identification *information* was collected. Given that the videos contained neutral, low-intensity emotional content, no significant distress was expected. The study was approved by the Ethics Committee of the Jerusalem Multidisciplinary College (IRB approval no. 0693; approved 11 November 2025) prior to data collection.

### 2.7. Statistical Analyses

We began our analyses by examining the Pearson product-moment correlation coefficients among the variables. This was followed by a series of conditional process analyses (i.e., moderated mediation analyses) because we expected the experimental condition (moderator) to moderate the associations that narcissistic admiration and narcissistic rivalry (predictors) had with perceived recognition (mediator), which, in turn, would be associated with the evaluations of the robot (outcomes). This analytic strategy was specified a priori to test theoretically distinct pathways associated with narcissistic admiration (H1–H2) and narcissistic rivalry (H3–H4), consistent with the Narcissistic Admiration and Rivalry Concept. Accordingly, separate conditional process models were estimated for each trait, each corresponding to a distinct set of hypotheses, rather than as exploratory or post hoc analyses. We were interested in each of the five subscales of the Robot Interaction Evaluation Scale (i.e., anthropomorphism, likability, perceived intelligence, safety, and intention to use), which prompted us to conduct separate conditional process analyses for each of these outcomes. Because we tested the same conditional process model across a family of theoretically related outcomes, we base our inferences primarily on the consistency of the indirect-effect pattern and on bootstrapped confidence intervals for conditional indirect effects and the index of moderated mediation, rather than on isolated p-values for individual outcomes.

We used model 8 of the PROCESS macro (Hayes, 2022) in conjunction with SPSS version 29 to conduct these conditional process analyses. PROCESS permits only a single focal predictor per model, with mediation and moderation applied exclusively to that variable. In our analyses, narcissistic admiration and narcissistic rivalry were therefore not included simultaneously as focal predictors within the same conditional process model. Rather, we estimated two conditional process models for each outcome, each with a different narcissism dimension specified as the focal predictor. In the first model, narcissistic admiration was specified as the focal predictor, with narcissistic rivalry included as a covariate along with its interaction with condition. In the second model, narcissistic rivalry was specified as the focal predictor, with narcissistic admiration included as a covariate along with its interaction with condition. Although covariates in PROCESS are not themselves subject to mediation or moderation, including the alternate narcissism dimension and its interaction with condition allowed us to statistically account for shared variance between narcissistic admiration and rivalry while isolating the conditional indirect effects of each focal predictor.

To ensure consistency across these models, we used a fixed seed command so that the bootstrap resampling procedure was identical across analyses. This approach yields linked estimates across models and allows for direct comparison of effects across the two narcissism dimensions. In this sense, although each conditional process model includes only a single focal predictor—as required by PROCESS—the analytic strategy permits estimation of effects for both narcissistic admiration and narcissistic rivalry in a coordinated and statistically coherent manner.

All of the variables were standardized to enhance the interpretability of the coefficients. We verified that the basic assumptions for these analyses were met prior to conducting these analyses (e.g., normally distributed residuals, homoscedasticity of the residuals, the absence of multicollinearity). For example, the Variance Inflation Factor (VIF) values were less than 1.21, which suggests that multicollinearity was not a concern. We conducted preliminary analyses that included gender as a moderator for exploratory purposes. However, gender did not emerge as a moderator in any of these analyses. As a result, we did not include gender in the final analyses, nor do we discuss gender differences in the interest of parsimony. Finally, although condition was experimentally manipulated, narcissistic admiration and rivalry were measured individual-difference variables. Accordingly, we interpret condition effects as causal inferences of the manipulation, but we refrain from causal claims regarding the associations involving narcissistic traits beyond the tested model structure.

### 2.8. Data Availability

Although this study was not pre-registered, to enhance transparency and reproducibility, the study materials are openly available on the Open Science Framework (OSF) at https://osf.io/92mpk. The repository includes: (a) the anonymized dataset used for analysis, (b) the final video stimuli files for both conditions and gender (4 files), and (c) the full text prompts/storyboard and production notes used to generate the stimuli.

## 3. Results

### 3.1. Background and Sociodemographic Variables

Table 1 presents the sociodemographic and background characteristics of the sample, shown overall and stratified by gender and experimental condition. The variables include age, family status, number of children, educational attainment, employment status, household income, religiosity, prior experience with robots, and brief indicators of loneliness and perceived social support. These variables are reported to describe the participants and to provide context for interpreting the experimental findings. Between-condition comparisons using t tests and χ^2^ tests revealed no significant differences between the cold robot and empathic robot conditions for any sociodemographic and background variable (all *p*s > 0.05). Notably, the groups were closely comparable on age, education, prior robot experience, and the background indicators most plausibly related to responses to a socially assistive robot (e.g., loneliness and perceived social support). This pattern supports the internal validity of the experimental comparison by reducing the likelihood that condition effects reflect pre-existing group differences. Accordingly, these variables were not included as covariates in the primary analyses.

### 3.2. Univariate Analyses

The correlation coefficients and descriptive statistics can be found in Table 2. Narcissistic admiration had small-to-medium correlations with narcissistic rivalry, perceived recognition, likability, and intention to use in both conditions. Narcissistic admiration had a small positive correlation with safety in the cold robot condition that did not emerge in the empathic robot condition, whereas it had positive correlations with anthropomorphism and perceived intelligence in the empathic condition that did not emerge in the cold robot condition. Narcissistic rivalry had a small positive association with the intention to use in both conditions. Interestingly, narcissistic rivalry had a small negative correlation with perceived recognition in the empathic robot condition, but this association reversed sign and became a small positive correlation in the cold robot condition. Narcissistic rivalry had a small negative correlation with perceived intelligence in the empathic robot condition, but this association did not emerge in the cold robot condition.

As shown in Table 3, participants in the cold robot condition did not differ from those in the empathic robot condition in terms of narcissistic admiration and rivalry. These results suggest that the random assignment of participants to the cold robot and empathic robot conditions led to the groups having similar levels of these traits. As expected, participants in the empathic robot condition reported higher levels of perceived recognition, anthropomorphism, and likability than those in the cold robot condition. However, no differences were found between the cold and empathic robot conditions for perceived intelligence, safety, and intention to use.

Notably, although mean-level comparisons revealed no condition differences in perceived intelligence, safety, or intention to use (see Table 3), condition effects emerged in the conditional process models once narcissistic admiration, narcissistic rivalry, and perceived recognition were included. These effects reflected differences in the direct impact of condition after accounting for narcissistic personality traits and perceived recognition, indicating that these variables played key roles in revealing condition-specific associations that were not evident in the mean-level analyses. This pattern suggests that once perceived recognition is statistically accounted for, the remaining (direct) effect of condition may reflect other components of the empathic style not captured by recognition; we return to this interpretive possibility in the Discussion.

### 3.3. Perceived Recognition

**Figure 3:**
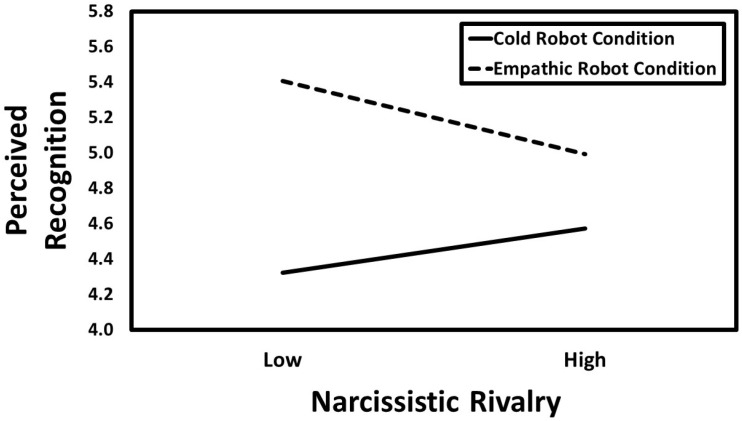
Predicted values illustrating the interaction that narcissistic rivalry had with experimental condition for perceived recognition.

The results of the conditional process analysis showed that narcissistic admiration was positively associated with perceived recognition (*B* = 0.20, *CI*_95%_[0.12, 0.28], *SE* = 0.04, *t* = 4.87, *p* < 0.001), whereas narcissistic rivalry was unrelated to perceived recognition (*B* = −0.04, *CI*_95%_[−0.12, 0.04], *SE* = 0.04, *t* = −0.88, *p* = 0.380). Experimental condition was also a significant predictor such that participants in the empathic robot condition reported higher perceived recognition than those in the cold robot condition (*B* = 0.34, *CI*_95%_[0.26, 0.42], *SE* = 0.04, *t* = 8.49, *p* < 0.001). The association between narcissistic admiration and perceived recognition was not moderated by experimental condition (*B* = 0.03, *CI*_95%_[−0.05, 0.11], *SE* = 0.04, *t* = 0.67, *p* = 0.504). In contrast, the narcissistic rivalry × experimental condition interaction was significant (*B* = −0.15, *CI*_95%_[−0.23, −0.07], *SE* = 0.04, *t* = −3.64, *p* < 0.001). Simple slope analyses indicated that narcissistic rivalry was negatively associated with perceived recognition in the empathic robot condition (*B* = −0.18, *CI*_95%_[−0.30, −0.07], *SE* = 0.06, *t* = −3.09, *p* = 0.002), whereas in the cold robot condition it showed a positive association (*B* = 0.11, *CI*_95%_[0.00, 0.22], *SE* = 0.06, *t* = 2.02, *p* = 0.044) See Figure 3.

Table 4, Table 5, Table 6, Table 7 and Table 8 are organized using a parallel reporting structure to facilitate interpretation across models. Each table includes (a) the mediator model predicting perceived recognition and (b) the outcome model predicting the specific robot-evaluation dimension. Because the mediator model is the same across these analyses, it is reproduced in Table 4, Table 5, Table 6, Table 7 and Table 8 for completeness, including *R*^2^ and the omnibus F test. The outcome models differ across tables only with respect to the specific evaluation dimension examined. Accordingly, the Results text focuses on hypothesis-relevant effects, including interaction terms where applicable, the bootstrapped indirect effects with 95% confidence intervals, and the index of moderated mediation.

### 3.4. Anthropomorphism

The results of the conditional process analysis for anthropomorphism are presented in Table 4. Neither narcissistic admiration (*B* = 0.04, *CI*_95%_[−0.04, 0.11], *SE* = 0.04, *t* = 0.95, *p* = 0.343) nor narcissistic rivalry (*B* = 0.00, *CI*_95%_[−0.08, 0.07], *SE* = 0.04, *t* = −0.13, *p* = 0.900) were associated with anthropomorphism. Experimental condition was also unrelated to anthropomorphism (*B* = 0.04, *CI*_95%_[−0.03, 0.12], *SE* = 0.04, *t* = 1.11, *p* = 0.270). In contrast, perceived recognition had a positive association with anthropomorphism (*B* = 0.52, *CI*_95%_[0.45, 0.60], *SE* = 0.04, *t* = 12.97, *p* < 0.001).

Supporting H1, narcissistic admiration demonstrated a positive indirect association with anthropomorphism through perceived recognition (*B* = 0.19, *CI*_95%_[0.10, 0.29], *SE* = 0.05, *z* = 4.38, *p* < 0.001). Contrary to H2, this indirect association was not moderated by experimental condition (*B* = 0.03, *CI*_95%_[−0.06, 0.12], *SE* = 0.05). In contrast, narcissistic rivalry did not exhibit an indirect association with anthropomorphism via perceived recognition (*B* = −0.03, *CI*_95%_[−0.11, 0.05], *SE* = 0.04, *z* = −0.64, *p* = 0.521), failing to support H3. Nevertheless, the index of moderated mediation was significant (*B* = −0.16, *CI*_95%_[−0.24, −0.08], *SE* = 0.04), as predicted in H4. More specifically, narcissistic rivalry had a negative indirect association with anthropomorphism through perceived recognition in the empathic robot condition (*B* = −0.10, *CI*_95%_[−0.15, −0.05], *SE* = 0.02) but not in the cold robot condition (*B* = 0.06, *CI*_95%_[0.00, 0.12], *SE* = 0.03).

**Table 4:** Results of the conditional process analysis for anthropomorphism.

	Outcome							
	M: Perceived Recognition				Y: Anthropomorphism			
Predictor	*Coeff.*	*SE*		*p*	*Coeff.*	*SE*		*p*
X_1_: Narcissistic Admiration (ADM)	0.20	0.04		<0.001	0.04	0.04		0.343
X_2_: Narcissistic Rivalry (RIV)	−0.04	0.04		0.380	0.00	0.04		0.900
M: Perceived Recognition	–	–		–	0.52	0.04		<0.001
W: Condition	0.34	0.04		<0.001	0.04	0.04		0.270
X_1_ × W: ADM × Condition	0.03	0.04		0.504	0.04	0.04		0.243
X_2_ × W: RIV × Condition	−0.15	0.04		<0.001	0.05	0.04		0.217
Constant	−0.10	0.04		0.813	0.00	0.04		0.989
	*R*^2^ = 0.17				*R*^2^ = 0.29			
	*F* = 22.03, *p* < 0.001				*F* = 36.84, *p* < 0.001			
Conditional Indirect Association of ADM with Anthropomorphism through Perceived Recognition								
Condition	*Coeff.*		*Boot SE*		*Boot LCI*		*Boot UCI*	
Cold (−1)	0.09		0.04		0.01		0.17	
Empathic (+1)	0.12		0.03		0.07		0.18	
Conditional Indirect Association of RIV with Anthropomorphism through Perceived Recognition								
Condition	*Coeff.*		*Boot SE*		*Boot LCI*		*Boot UCI*	
Cold (−1)	0.06		0.03		0.00		0.12	
Empathic (+1)	−0.10		0.02		−0.15		−0.05	

### 3.5. Likability

The results of the conditional process analysis for likability are presented in Table 5. Neither narcissistic admiration (*B* = 0.05, *CI*_95%_[−0.03, 0.12], *SE* = 0.04, *t* = 1.26, *p* = 0.207) nor narcissistic rivalry (*B* = −0.04, *CI*_95%_[−0.11, 0.03], *SE* = 0.04, *t* = −1.10, *p* = 0.270) were associated with likability. Experimental condition was also unrelated to likability (*B* = 0.03, *CI*_95%_[−0.05, 0.10], *SE* = 0.04, *t* = 0.68, *p* = 0.496). In contrast, perceived recognition had a positive association with likability (*B* = 0.60, *CI*_95%_[0.53, 0.68], *SE* = 0.04, *t* = 15.85, *p* < 0.001).

Consistent with H1, narcissistic admiration demonstrated a positive indirect association with likability through perceived recognition (*B* = 0.19, *CI*_95%_[0.10, 0.29], *SE* = 0.05, *z* = 4.45, *p* < 0.001). However, this indirect association was not moderated by experimental condition (*B* = 0.03, *CI*_95%_[−0.07, 0.14], *SE* = 0.05), which is contrary to H2. In contrast, narcissistic rivalry did not exhibit an indirect association with likability via perceived recognition(*B* = −0.03, *CI*_95%_[−0.11, 0.05], *SE* = 0.05, *z* = −0.64, *p* = 0.521), failing to support H3. Nevertheless, the index of moderated mediation was significant (*B* = −0.18, *CI*_95%_[−0.27, −0.09], *SE* = 0.05), as predicted in H4. More specifically, narcissistic rivalry had a negative indirect association with likability through perceived recognition in the empathic robot condition (*B* = −0.11, *CI*_95%_[−0.17, −0.06], *SE* = 0.03) but not in the cold robot condition (*B* = 0.07, *CI*_95%_[0.00, 0.14], *SE* = 0.04).

**Table 5:** Results of the conditional process analysis for likability.

	Outcome							
	M: Perceived Recognition				Y: Likability			
Predictor	*Coeff.*	*SE*		*p*	*Coeff.*	*SE*		*p*
X_1_: Narcissistic Admiration (ADM)	0.20	0.04		<0.001	0.05	0.04		0.207
X_2_: Narcissistic Rivalry (RIV)	−0.04	0.04		0.380	−0.00	0.04		0.270
M: Perceived Recognition	–	–		–	0.60	0.04		<0.001
W: Condition	0.34	0.04		<0.001	0.03	0.04		0.496
X_1_ × W: ADM × Condition	0.03	0.04		0.504	−0.03	0.04		0.432
X_2_ × W: RIV × Condition	−0.15	0.04		<0.001	0.02	0.04		0.563
Constant	−0.10	0.04		0.813	0.00	0.03		0.987
	*R*^2^ = 0.17				*R*^2^ = 0.38			
	*F* = 22.03, *p* < 0.001				*F* = 53.67, *p* < 0.001			
Conditional Indirect Association of ADM with Likability through Perceived Recognition								
Condition	*Coeff.*		*Boot SE*		*Boot LCI*		*Boot UCI*	
Cold (−1)	0.10		0.05		0.01		0.20	
Empathic (+1)	0.14		0.03		0.08		0.20	
Conditional Indirect Association of RIV with Likability through Perceived Recognition								
Condition	*Coeff.*		*Boot SE*		*Boot LCI*		*Boot UCI*	
Cold (−1)	0.07		0.04		0.00		0.14	
Empathic (+1)	−0.11		0.03		−0.17		−0.06	

### 3.6. Perceived Intelligence

The results of the conditional process analysis for perceived intelligence are presented in Table 6. Narcissistic rivalry was negatively associated with perceived intelligence (*B* = −0.09, *CI*_95%_[−0.17, −0.01], *SE* = 0.04, *t* = −2.09, *p* = 0.037), whereas narcissistic admiration was not associated with perceived intelligence (*B* = 0.03, *CI*_95%_[−0.05, 0.11], *SE* = 0.04, *t* = 0.71, *p* = 0.477). Experimental condition was negatively associated with perceived intelligence such that participants in the cold robot condition evaluated the robot as more intelligent than those in the empathic robot condition (*B* = −0.17, *CI*_95%_[−0.25, −0.09], *SE* = 0.04, *t* = −3.91, *p* < 0.001). In contrast, perceived recognition had a positive association with the perceived intelligence of the robot (*B* = 0.43, *CI*_95%_[0.34, 0.51], *SE* = 0.04, *t* = 9.82, *p* < 0.001).

Supporting H1, narcissistic admiration demonstrated a positive indirect association with perceived intelligence through perceived recognition (*B* = 0.10, *CI*_95%_[0.05, 0.15], *SE* = 0.02, *z* = 4.10, *p* < 0.001). Contrary to H2, this indirect association was not moderated by experimental condition (*B* = 0.02, *CI*_95%_[−0.05, 0.10], *SE* = 0.04). In contrast, narcissistic rivalry did not exhibit an indirect association with perceived intelligence via perceived recognition (*B* = −0.02, *CI*_95%_[−0.06, 0.03], *SE* = 0.02, *z* = −0.64, *p* = 0.523), failing to provide support for H3. However, the index of moderated mediation was significant (*B* = −0.13, *CI*_95%_[−0.20, −0.06], *SE* = 0.03), as predicted by H4. More specifically, narcissistic rivalry had a negative indirect association with perceived intelligence through perceived recognition in the empathic robot condition (*B* = −0.08, *CI*_95%_[−0.12, −0.04], *SE* = 0.02) but not in the cold robot condition (*B* = 0.05, *CI*_95%_[0.00, 0.10], *SE* = 0.03).

**Table 6:** Results of the conditional process analysis for perceived intelligence.

	Outcome							
	M: Perceived Recognition				Y: Perceived Intelligence			
Predictor	*Coeff.*	*SE*		*p*	*Coeff.*	*SE*		*p*
X_1_: Narcissistic Admiration (ADM)	0.20	0.04		<0.001	0.03	0.04		0.477
X_2_: Narcissistic Rivalry (RIV)	−0.04	0.04		0.380	−0.09	0.04		0.037
M: Perceived Recognition	–	–		–	0.43	0.04		<0.001
W: Condition	0.34	0.04		<0.001	−0.17	0.04		<0.001
X_1_ × W: ADM × Condition	0.03	0.04		0.504	0.05	0.04		0.191
X_2_ × W: RIV × Condition	−0.15	0.04		<0.001	−0.03	0.04		0.444
Constant	−0.10	0.04		0.813	0.00	0.04		0.918
	*R*^2^ = 0.17				*R*^2^ = 0.18			
	*F* = 22.03, *p* < 0.001				*F* = 19.19, *p* < 0.001			
Conditional Indirect Association of ADM with Perceived Intelligence through Perceived Recognition								
Condition	*Coeff.*		*Boot SE*		*Boot LCI*		*Boot UCI*	
Cold (−1)	0.07		0.03		0.01		0.14	
Empathic (+1)	0.10		0.02		0.05		0.15	
Conditional Indirect Association of RIV with Perceived Intelligence through Perceived Recognition								
Condition	*Coeff.*		*Boot SE*		*Boot LCI*		*Boot UCI*	
Cold (−1)	0.05		0.03		0.00		0.10	
Empathic (+1)	−0.08		0.02		−0.12		−0.04	

### 3.7. Safety

The results of the conditional process analysis for safety are presented in Table 7. Neither narcissistic admiration (*B* = 0.05, *CI*_95%_[−0.03, 0.14], *SE* = 0.04, *t* = 1.22, *p* = 0.224) nor narcissistic rivalry (*B* = 0.02, *CI*_95%_[−0.06, 0.10], *SE* = 0.04, *t* = 0.53, *p* = 0.600) were associated with safety. Experimental condition was negatively associated with safety such that participants in the cold robot condition evaluated the robot as safer than those in the empathic robot condition (*B* = −0.11, *CI*_95%_[−0.20, −0.02], *SE* = 0.04, *t* = −2.53, *p* = 0.012). In contrast, perceived recognition had a positive association with the safety of the robot (*B* = 0.37, *CI*_95%_[0.29, 0.46], *SE* = 0.05, *t* = 8.25, *p* < 0.001).

Narcissistic admiration demonstrated a positive indirect association with safety through perceived recognition (*B* = 0.10, *CI*_95%_[0.05, 0.16], *SE* = 0.02, *z* = 3.95, *p* < 0.001), which supports H1. This indirect association was not moderated by experimental condition (*B* = 0.02, *CI*_95%_[−0.05, 0.09], *SE* = 0.03), failing to support H2. Contrary to H3, narcissistic rivalry did not exhibit an indirect association with safety via perceived recognition (*B* = −0.02, *CI*_95%_[−0.06, 0.03], *SE* = 0.02, *z* = −0.64, *p* = 0.525). However, the index of moderated mediation was significant (*B* = −0.11, *CI*_95%_[−0.18, −0.05], *SE* = 0.03), which supports H4. More specifically, narcissistic rivalry had a negative indirect association with safety through perceived recognition in the empathic robot condition (*B* = −0.07, *CI*_95%_[−0.11, −0.03], *SE* = 0.02) but not in the cold robot condition (*B* = 0.04, *CI*_95%_[0.00, 0.09], *SE* = 0.02).

**Table 7:** Results of the conditional process analysis for safety.

	Outcome							
	M: Perceived Recognition				Y: Safety			
Predictor	*Coeff.*	*SE*		*p*	*Coeff.*	*SE*		*p*
X_1_: Narcissistic Admiration (ADM)	0.20	0.04		<0.001	0.05	0.04		0.224
X_2_: Narcissistic Rivalry (RIV)	−0.04	0.04		0.380	0.02	0.04		0.600
M: Perceived Recognition	–	–		–	0.37	0.05		<0.001
W: Condition	0.34	0.04		<0.001	−0.11	0.04		0.012
X_1_ × W: ADM × Condition	0.03	0.04		0.504	−0.02	0.04		0.664
X_2_ × W: RIV × Condition	−0.15	0.04		<0.001	0.04	0.04		0.318
Constant	−0.10	0.04		0.813	0.00	0.04		0.942
	*R*^2^ = 0.17				*R*^2^ = 0.13			
	*F* = 22.03, *p* < 0.001				*F* = 13.03 *p* < 0.001			
Conditional Indirect Association of ADM with Safety through Perceived Recognition								
Condition	*Coeff.*		*Boot SE*		*Boot LCI*		*Boot UCI*	
Cold (−1)	0.06		0.03		0.01		0.13	
Empathic (+1)	0.08		0.02		0.05		0.13	
Conditional Indirect Association of RIV with Safety through Perceived Recognition								
Condition	*Coeff.*		*Boot SE*		*Boot LCI*		*Boot UCI*	
Cold (−1)	0.04		0.02		0.00		0.09	
Empathic (+1)	−0.07		0.02		−0.11		−0.03	

### 3.8. Intention to Use

The results of the conditional process analysis for intention to use are presented in Table 8. Narcissistic rivalry was positively associated with intention to use (*B* = 0.14, *CI*_95%_[0.06, 0.22], *SE* = 0.04, *t* = 3.42, *p* < 0.001), whereas narcissistic admiration was not associated with intention to use (*B* = 0.06, *CI*_95%_[−0.02, 0.15], *SE* = 0.04, *t* = 1.45, *p* = 0.149). Experimental condition was negatively associated with intention to use such that participants in the cold robot condition reported a greater intention to use the robot than those in the empathic robot condition (*B* = −0.14, *CI*_95%_[−0.22, −0.05], *SE* = 0.04, *t* = −3.17, *p* = 0.002). In contrast, perceived recognition had a positive association with the intention to use the robot (*B* = 0.34, *CI*_95%_[0.25, 0.43], *SE* = 0.04, *t* = 7.61, *p* < 0.001).

Supporting H1, narcissistic admiration demonstrated a positive indirect association with intention to use the robot through perceived recognition (B = 0.12, *CI*_95%_[0.06, 0.20], SE = 0.03, z = 3.77, *p* < 0.001). Contrary to H2, this indirect association was not moderated by experimental condition (B = 0.02, *CI*_95%_[−0.04, 0.08], SE = 0.03). In contrast, narcissistic rivalry did not exhibit an indirect association with the intention to use the robot via perceived recognition (B = −0.02, *CI*_95%_[−0.08, 0.03], SE = 0.03, z = −0.63, *p* = 0.526), failing to support H3. Nevertheless, the index of moderated mediation was significant (B = −0.10, *CI*_95%_[−0.16, −0.05], SE = 0.03), as predicted in H4. More specifically, narcissistic rivalry had a negative indirect association with perceived intention to use through perceived recognition in the empathic robot condition (B = −0.06, *CI*_95%_[−0.10, −0.03], SE = 0.02) but not in the cold robot condition (B = 0.04, *CI*_95%_[0.00, 0.08], SE = 0.02).

**Table 8:** Results of the conditional process analysis for intention to use.

	Outcome							
	M: Perceived Recognition				Y: Intention to Use			
Predictor	*Coeff.*	*SE*		*p*	*Coeff.*	*SE*		*p*
X_1_: Narcissistic Admiration (ADM)	0.20	0.04		<0.001	0.06	0.04		0.149
X_2_: Narcissistic Rivalry (RIV)	−0.04	0.04		0.380	0.14	0.04		<0.001
M: Perceived Recognition	–	–		–	0.34	0.04		<0.001
W: Condition	0.34	0.04		<0.001	−0.14	0.04		0.002
X_1_ × W: ADM × Condition	0.03	0.04		0.504	0.02	0.04		0.585
X_2_ × W: RIV × Condition	−0.15	0.04		<0.001	0.07	0.04		0.078
Constant	−0.10	0.04		0.813	0.00	0.04		0.920
	*R*^2^ = 0.17				*R*^2^ = 0.14			
	*F* = 22.03, *p* < 0.001				*F* = 13.86, *p* < 0.001			
Conditional Indirect Association of ADM with Intention to Use through Perceived Recognition								
Condition	*Coeff.*		*Boot SE*		*Boot LCI*		*Boot UCI*	
Cold (−1)	0.06		0.03		0.01		0.12	
Empathic (+1)	0.08		0.02		0.04		0.12	
Conditional Indirect Association of RIV with Intention to Use through Perceived Recognition								
Condition	*Coeff.*		*Boot SE*		*Boot LCI*		*Boot UCI*	
Cold (−1)	0.04		0.02		0.00		0.08	
Empathic (+1)	−0.06		0.02		−0.10		−0.03	

## 4. Discussion

### 4.1. Summary of the Main Findings

The present study examined a recognition-based “mirror effect” framework in which older adults’ narcissistic admiration and rivalry were hypothesized to be associated with evaluations of a SAR indirectly via perceived recognition, with robot behavioral style (empathic vs. cold) functioning as a contextual moderator.

#### Hypotheses and Overall Pattern of Support

The results provided clear support for the core mediation component of the model. Consistent with H1, narcissistic admiration was positively associated with perceived recognition, which, in turn, predicted more favorable evaluations of the robot. However, support was not found for the predicted moderation of this indirect association (H2); the indirect association linking narcissistic admiration to evaluations of the robot via perceived recognition did not reliably vary as a function of the interaction style of the robot.

In contrast, narcissistic rivalry exhibited a condition-dependent recognition pathway. Higher levels of narcissistic rivalry were associated with lower perceived recognition and, in turn, less favorable evaluations of the robot, but this pattern emerged primarily in the empathic robot condition. In the cold robot condition, the corresponding indirect association was absent or substantially weaker. Thus, H3 received conditional support, whereas H4 was not supported in the predicted direction.

Importantly, the “mirror effect” framework refers to the recognition-based mechanism itself—that is, the indirect pathway from personality to evaluation via perceived recognition—rather than to a fixed expectation that empathic cues will uniformly attenuate rivalry-related negativity. Instead, the observed moderation pattern highlights a meaningful boundary condition: the rivalry → recognition → evaluation pathway was more pronounced under empathic interaction styles than under cold ones.

This directional reversal is theoretically informative, as it suggests that heightened relational signaling may elicit threat-related or evaluative interpretations among rivalry-prone users, thereby reducing perceived recognition and amplifying negative evaluations rather than mitigating them.

Three core findings warrant emphasis. First, empathic robot behavior increased perceived recognition and also elevated anthropomorphism and likability at the mean level. This pattern is consistent with prior evidence that socio-emotional cues (e.g., prosody, gaze, responsive feedback) enhance perceptions of warmth, social presence, and acceptance in HRI (Reeves & Nass, 1996; Park & Whang, 2022; Stock-Homburg, 2021; Short et al., 1976). Second, perceived recognition emerged as a robust and consistent predictor of more favorable robot evaluations across multiple domains commonly used in HRI research (Bartneck et al., 2008). This finding suggests that feeling “seen” and acknowledged may represent a central psychological mechanism through which interactional cues translate into downstream judgments of artificial agents. Third, narcissistic admiration showed reliable positive indirect associations with robot evaluations through perceived recognition, indicating that admiration-oriented self-regulation is linked to extracting or construing validating meaning from the interaction (Back et al., 2013; Morf & Rhodewalt, 2001). Notably, contrary to our expectations, admiration’s indirect associations were not stronger in the empathic robot condition compared to the cold robot condition.

By contrast, narcissistic rivalry displayed a markedly condition-dependent pattern. In the empathic condition, higher levels of narcissistic rivalry predicted lower perceived recognition and, in turn, less favorable evaluations. In the cold condition, narcissistic rivalry was not associated with diminished recognition and showed small positive conditional indirect associations for some outcomes. This pattern challenges the assumption that empathic behavior is uniformly beneficial and suggests that, for individuals characterized by defensive self-protection, heightened relational signaling may evoke threat-related interpretations that reduce perceived recognition and devalue the agent (Back et al., 2013; Morf & Rhodewalt, 2001). This pattern aligns with the argument that empathic robotic responses may provoke negative responses in some users (e.g., discomfort, feelings of inauthenticity; Fuchs, 2024).

Taken together, these findings suggest that recognition-based mechanisms are robust across robot style, whereas rivalry-related threat sensitivity may be especially triggered by empathic cues that are experienced as evaluative or inauthentic.

### 4.2. Theoretical Contributions: Recognition as a Central Mechanism in HRI

These findings extend theory in HRI by positioning perceived recognition as a central mechanism connecting robot behavior and user evaluations. Extensive research shows that people readily apply social norms to machines, even with minimal cues, supporting the idea that artificial agents can become psychologically meaningful social partners (Reeves & Nass, 1996). Prior work has emphasized warmth, social presence, and anthropomorphism as proximal drivers of acceptance (Bartneck et al., 2008; Stock-Homburg, 2021). The present results add an integrative layer by suggesting that it is not merely “warmth” as a surface impression that matters, but the deeper experience of being acknowledged and valued. Conceptually, this aligns HRI with recognition-based perspectives in social philosophy, in which recognition is tied to dignity, identity, and social worth (Fraser, 2000; Honneth, 1996). From a psychological standpoint, recognition is also closely connected to belongingness motives (Baumeister & Leary, 1995), which may be particularly relevant in older adulthood, when social isolation is a salient risk factor (Isabet et al., 2021). In this sense, perceived recognition may function as a bridge construct, linking design-level cues (e.g., empathic speech, gaze) to human needs for social affirmation, while remaining sensitive to individual differences that shape whether such cues are experienced as supportive or intrusive.

Importantly, perceived recognition is not intended as a re-labeling of warmth, social presence, or anthropomorphism. Whereas warmth and social presence describe the general socio-emotional tone and vividness of an interaction, and anthropomorphism reflects the extent to which the robot is perceived as human-like, recognition captures a more self-referential experience, feeling that the agent is attending to *me* as a particular person (i.e., being seen, acknowledged, and valued). Framed this way, recognition functions as a process mechanism that can explain how design-level cues translate into downstream evaluations, rather than as simply another evaluative outcome. Empirically, perceived recognition was strongly related to anthropomorphism and likability (see Table 2), yet the associations were clearly below unity, supporting the view that recognition is related but not redundant with these constructs.

### 4.3. Personality Dynamics: Why Admiration Benefits and Rivalry Can Backfire

The narcissistic admiration findings are consistent with the Narcissistic Admiration and Rivalry Concept (NARC), which distinguishes a self-enhancement pathway (admiration) from a defensive antagonistic pathway (rivalry; Back et al., 2013). For narcissistic admiration, recognition cues may be readily construed as validating feedback, thereby increasing perceived recognition and downstream positivity toward the robot (Morf & Rhodewalt, 2001). The absence of moderation for admiration suggests a robust “recognition-seeking” tendency that operates across contexts: even when the robot is colder, individuals high in narcissistic admiration may still interpret the interaction in self-affirming ways or selectively attend to cues that support a positive self-view.

The narcissistic rivalry pattern is theoretically more revealing. Narcissistic rivalry is characterized by vigilance to ego threat and a tendency to devalue others when status is challenged (Back et al., 2013). From a NARC perspective, this finding suggests that empathic behavior does not merely provide “more recognition” but may also increase the diagnosticity of interpersonal cues. In an empathic interaction, the robot’s relational cues (e.g., eye contact, supportive language) may paradoxically intensify evaluative salience, making the exchange feel more “personal,” thereby increasing opportunities for threat detection, suspicion, or concerns about authenticity. For some older adults, highly empathic scripts may also be experienced as patronizing or autonomy-reducing (i.e., ‘over-involvement’), which can undermine dignity even when the intention is supportive. This framing helps explain why rivalry-prone individuals, who are especially vigilant to status and control cues, may respond more negatively to relationally rich empathic signaling than to a respectful-neutral (cold) style.

While prior work suggests that overly empathic robot behavior may be experienced as simulated or unsettling (Fuchs, 2024), the present findings indicate that such reactions are not uniform, but are contingent on users’ narcissistic self-regulation. Specifically, empathic cues appear to increase the interpretive weight of the interaction, such that for individuals high in narcissistic rivalry, heightened relational signaling amplifies threat sensitivity and suspicion rather than perceived recognition. In other words, the robot is not the source of the problem; it is its mirror that exposes an existing psychological dynamic.

From this view, empathic cues may not always translate into recognition (i.e., “more empathy = more recognition”); for rivalry-prone users, the same cues may be interpreted as inauthentic or controlling, reducing the felt experience of being genuinely acknowledged. In contrast, the cold condition may present fewer relational cues to appraise, thereby reducing the likelihood that rivalry-linked threat sensitivity is activated. Thus, the observed reversal of H4 highlights a boundary condition: relationally rich, empathic signaling can be beneficial for many users yet counterproductive for those prone to rivalry-related threat appraisals. This interpretation is consistent with recent work emphasizing that intelligent agents can participate in morally and relationally charged exchanges, shaping users’ trust and perceived intentions beyond functional performance (D’Angelo et al., 2023; Sgorbissa et al., 2024).

### 4.4. Reconciling Mean Differences with Model-Based Condition Effects

An important nuance concerns condition effects that appear differently across analytic lenses. At the mean level, the empathic condition increased perceived recognition, anthropomorphism, and likability, whereas perceived intelligence, safety, and intention to use did not differ significantly across conditions. However, in the conditional process models, condition showed negative direct effects for some outcomes when perceived recognition was included. This pattern is consistent with a suppression-like situation: empathic behavior increases recognition (which strongly predicts favorable outcomes), yet once that recognition pathway is accounted for, residual aspects of the empathic style (e.g., perceived artificiality, over-involvement, or boundary concerns) may reduce some evaluations for certain users. Substantively, this reinforces the central theoretical message: recognition is a key benefit channel of empathic HRI, but empathic cues may also carry costs that become visible when the “recognition benefit” is statistically separated from other components of the interaction. Future work should directly test these candidate costs by measuring perceived authenticity, intrusiveness, privacy concerns, and perceived manipulation alongside recognition.

### 4.5. Practical Implications for Social-Robot Design in Older Adult Care

The findings have direct implications for the design, development and deployment of SAR. First, they suggest that perceived recognition should be treated as a core interaction target rather than a byproduct of empathic cues alone. From a design and development perspective, this implies that robot interfaces should prioritize mechanisms that make users feel seen, acknowledged, and responded to by contingent turn-taking, adaptive timing, personalized address, and transparent responsiveness. Computationally, this suggests moving beyond static empathic scripts toward recognition-sensitive interaction logic, in which user inputs and behaviors are explicitly acknowledged in ways that are interpretable to the user.

Second, the findings regarding narcissistic rivalry caution against a one-size-fits-all empathy model. While empathic behavior is often assumed to be universally beneficial in elder-care robotics, our results demonstrate that heightened relational signaling may be counterproductive for some users. This has important implications for system design and deployment: rather than defaulting to maximal empathy, SARs should support multiple interaction modes. From a developer standpoint, this can be operationalized through configurable behavioral profiles, for example, a *respectful-neutral mode* emphasizing clarity, competence, and predictability, alongside an *empathic mode* that includes affective language, relational cues, and emotional affirmation. Care staff and system operators should be trained to select or adjust these modes based on observed user responses rather than assuming empathy as the optimal default (de Graaf & Ben Allouch, 2013; Fuchs, 2024).

Taken together, these implications suggest that effective SAR deployment requires adaptive interaction architectures that balance empathic expressiveness with user-specific sensitivity, combining design decisions, behavioral logic, and deployment protocols. Such systems should be capable of dynamically calibrating relational intensity while maintaining transparency and user autonomy, an approach that is both technically feasible and ethically aligned with older adult care contexts.

### 4.6. Ethical and Psychological Considerations of Recognition-Based AI

These practical insights also raise broader ethical and psychological questions about the implications of recognition-based AI. Recognition is deeply tied to dignity and identity (Fraser, 2000; Honneth, 1996), and robots designed to provide acknowledgment may support belongingness needs (Baumeister & Leary, 1995), potentially benefiting older adults vulnerable to isolation (Isabet et al., 2021). Yet recognition delivered by SAR can also risk externalizing self-worth or fostering unrealistic expectations of reciprocity, especially when users become reliant on simulated validation (Fuchs, 2024). The present findings highlight that robots may participate in users’ self-regulatory processes while amplifying self-enhancement for admiration and potentially triggering threat dynamics for rivalry. Ethical design should therefore prioritize transparency, autonomy, and boundaries: robots can be supportive without implying mutuality, and systems should avoid manipulative social cues that might intensify dependency or distress (D’Angelo et al., 2023; Sgorbissa et al., 2024).

From a design standpoint, an important safeguard is to prevent recognition-oriented behaviors from becoming a persistent source of validation seeking. One practical guideline is to make empathic and recognition cues explicitly configurable and reversible, allowing users to opt into, attenuate, or disable relational signaling without penalty. A second guideline concerns transparency: supportive statements should be framed as assistance-oriented and autonomy-supportive rather than as evaluative praise, avoiding language that positions the robot as an authority conferring worth to the user. A third guideline is the adoption of a neutral-but-respectful default interaction style that communicates competence and respect while minimizing intensive emotional mirroring—particularly for users who may experience warmth as intrusive or evaluative. Together, these safeguards can reduce the risk of emotional dependency while preserving user dignity and agency.

In summary, the mirror-effect framework not only clarifies how individual differences shape responses to empathic HRI but also underscores ethical imperatives for responsible, recognition-sensitive SAR design. These findings raise broader questions about emotional authenticity and the boundary between simulated and genuine care. In particular, they motivate a critical ethical concern regarding “imposed empathy”: when simulated warmth is experienced as intrusive or evaluative, it may undermine dignity and autonomy rather than promote well-being. Accordingly, an ethically conservative design stance is to treat neutral-but-respectful interaction profiles as a legitimate default, while viewing more emotionally expressive styles as opt-in, user-governed features rather than universally beneficial defaults. Future research should therefore examine how empathic SAR and AI systems can balance responsiveness with user autonomy, ensuring that recognition-oriented design supports dignity rather than dependency.

### 4.7. Limitations

Several limitations warrant attention. First, participants responded to video-based vignettes rather than engaging in live, reciprocal interactions. Although this approach increases experimental control and is common in HRI research, real interactions may elicit more nuanced affective and behavioral responses. Second, most focal constructs were assessed via self-report immediately after the video, which may inflate associations due to common-method variance or halo effects. To address this concern, we conducted post hoc CFA-based diagnostics in AMOS (two-factor vs. one-factor models and a common latent factor specification; see Appendix B). These tests provide evidence against severe CMV (i.e., a single common factor could not account for the observed covariation), but they cannot rule out all sources of shared-method or halo effects; thus, the findings should be interpreted as robust to major CMV explanations rather than as proof of its absence. Third, the study was conducted online and partially relied on a volunteer panel; older adults who enroll in such panels may be higher in digital literacy or more open to technology than the broader older population, potentially limiting generalizability. Fourth, we did not include a direct participant-level manipulation check of the robot’s behavioral style (empathic vs. cold), as such a check could have primed participants and overlapped conceptually with our focal mediator (perceived recognition). Instead, we validated the intended contrast in expressed empathy using independent expert-judge ratings of the stimulus videos. Thus, we view the expert-judge evidence as supporting the manipulation’s construct validity while acknowledging that it cannot fully replace participants’ own appraisals during the study. Future work could incorporate a brief, unobtrusive end-of-survey assessment (e.g., 2–3 warmth/attunement items or comprehension-based checks) that avoids conceptual overlap with perceived recognition, to further strengthen internal validity. Fifth, the sample consisted of community-dwelling older adults from a specific cultural context, which may constrain generalizability. Sixth, the stimuli were AI-generated using multiple platforms with post-processing steps; even when prompts and parameters are held constant, subtle rendering differences across generators could introduce unintended variability. Finally, although established HRI evaluation measures were used (Bartneck et al., 2008), the Perceived Recognition Scale was newly developed for this study. Additional validation, such as demonstrating convergent associations with related constructs including belongingness, autonomy, perceived authenticity, or intrusiveness, would strengthen its interpretability and support its broader use in future research.

### 4.8. Directions for Future Research

Future studies should examine how perceived recognition unfolds dynamically across repeated or adaptive interactions with SAR. Longitudinal and in-the-wild designs would be particularly well suited to testing whether perceived recognition predicts downstream outcomes such as actual usage, self-disclosure, adherence, and well-being over time (Isabet et al., 2021). Experimental refinements could systematically manipulate degrees of empathic expression (e.g., low, moderate, high) and more clearly distinguish “neutral-but-respectful” interaction styles from genuinely “cold” ones, thereby clarifying whether rivalry-related effects stem from perceived intrusiveness rather than from a simple absence of warmth.

Research should also investigate additional moderators likely to interact with recognition processes in older adulthood, including loneliness, attachment orientation, prior attitudes toward robots, and perceived authenticity (de Graaf & Ben Allouch, 2013; Esterwood et al., 2021; Fuchs, 2024). Given that the present study was conducted in a Hebrew-speaking Israeli context, cross-cultural replications across both collectivistic and individualistic cultures are an essential next step for testing the generalizability of the mirror-effect model and for identifying potential cultural variability in narcissistic self-regulatory expressions.

Gender represents another important moderator for future work. Because the present design used gender-matched protagonists to support identification, it would be especially important for future studies to examine whether admiration- or rivalry-linked responses to empathic versus cold styles vary as a function of gendered social norms and expectations. Such research would inform more psychologically sensitive personalization strategies for SAR. Finally, integrating perspectives from ethical AI and recognition theory may help further specify design principles that maximize supportive recognition while minimizing dependency and preserving dignity (Fraser, 2000; Honneth, 1996).

## 5. Conclusions

The present study highlights perceived recognition as a central mechanism linking robot behavior, personality dynamics, and older adults’ evaluations of SAR. Narcissistic admiration showed consistent positive indirect associations with evaluations via perceived recognition, whereas narcissistic rivalry exhibited a condition-dependent pattern that challenges the assumption that empathic cues are uniformly beneficial. Together, these findings advance theory on personality–technology fit (Back et al., 2013; Morf & Rhodewalt, 2001) and underscore an applied message for elder-care robotics: recognition-sensitive design should be flexible and autonomy-respecting, calibrating socio-emotional expressiveness to users’ motivational profiles rather than assuming maximal empathy is always optimal.

## Figures and Tables

**Table 1:** Sociodemographic and background information.

		Men (*n* = 271)		Women (*n* = 256)		
		Experimental Condition				
	Total Sample (N = 527)	Cold Robot (*n* = 131)	Empathic Robot (*n* = 140)	Cold Robot (*n* = 126)	Empathic Robot (*n* = 130)	Cold ≠ Empathic Statistics
Age	72.73	73.21	74.03	71.84	71.71	*t* = −0.92
Number of children	2.90	2.96	2.98	2.76	2.88	*t* = −0.55
Sense of loneliness	22.58	23.98	19.30	25.11	22.25	*t* = 1.66
Perceived social support	73.41	71.40	74.46	74.69	73.06	*t* = −0.36
Previous experience with robots						*χ*^2^ = 0.15
Yes	41.2%	41.2%	45.0%	42.9%	35.4%	
No	58.8%	58.8%	55.0%	57.1%	64.6%	
Education						*χ*^2^ = 2.50
No high school degree	11.0%	9.2%	9.3%	15.9%	10.0%	
High school degree	27.7%	22.1%	27.9%	29.4%	31.5%	
Bachelor’s degree	36.4%	41.2%	32.9%	31.0%	40.8%	
Master’s degree	21.6%	24.4%	25.7%	21.4%	14.6%	
Ph.D. or equivalent	3.2%	3.1%	4.3%	2.4%	3.1%	
Employment						*χ*^2^ = 4.78
Full time	17.8%	21.4%	18.6%	18.3%	13.1%	
Part time	14.0%	11.5%	9.3%	16.7%	19.2%	
Unemployed	1.6%	1.6%	1.4%	0.8%	2.3%	
Going to school	0.6%	0.0%	0.0%	1.6%	0.8%	
Home maker	64.1%	62.6%	70.0%	60.3%	63.1%	
Retired	1.9%	3.1%	0.7%	2.4%	1.5%	
Marital Status						*χ*^2^ = 7.38
Single	2.8%	2.3%	0.7%	5.6%	3.1%	
Dating	0.8%	0.0%	0.0%	1.6%	1.6%	
Cohabiting	2.5%	3.8%	0.7%	3.2%	2.3%	
Married	71.7%	82.4%	85.0%	53.2%	64.6%	
Separated	0.6%	0.8%	0.0%	0.8%	0.8%	
Divorced	13.9%	8.4%	5.7%	22.2%	20.0%	
Widowed	7.8%	2.3%	7.9%	13.5%	7.7%	
Household income						*χ*^2^ = 3.19
Very high	12.9%	15.3%	20.0%	6.3%	9.2%	
Somewhat high	25.0%	32.1%	27.9%	19.0%	20.8%	
Moderate	32.3%	35.1%	30.7%	29.4%	33.8%	
Somewhat low	18.0%	6.9%	12.9%	28.6%	24.6%	
Very low	11.8%	10.7%	8.6%	16.7%	11.5%	
Religiosity						*χ*^2^ = 3.05
Secular	68.1%	72.5%	66.4%	69.0%	64.6%	
Traditional	20.1%	13.0%	26.4%	17.5%	23.1%	
Religious	8.2%	9.9%	5.7%	8.7%	8.5%	
Ultra-Orthodox	3.6%	4.6%	1.4%	4.8%	3.8%	

**Table 2:** Intercorrelations and Descriptive Statistics.

	1	2	3	4	5	6	7	8
1. Narcissistic Admiration	–	0.17 **	0.25 ***	0.19 **	0.15 *	0.15 *	0.11	0.18 **
2. Narcissistic Rivalry	0.24 ***	–	−0.18 **	−0.02	−0.11	−0.16 *	0.02	0.17 **
3. Perceived Recognition	0.19 **	0.15 *	–	0.44 ***	0.50 ***	0.45 ***	0.28 ***	0.30 ***
4. Anthropomorphism	0.09	0.03	0.57 ***	–	0.49 ***	0.48 ***	0.52 ***	0.52 ***
5. Likability	0.17 **	0.05	0.64 ***	0.64 ***	–	0.65 ***	0.59 ***	0.44 ***
6. Perceived Intelligence	0.05	0.01	0.40 ***	0.39 ***	0.61 ***	–	0.57 ***	0.43 ***
7. Safety	0.15 *	0.06	0.43 ***	0.40 ***	0.57 ***	0.60 ***	–	0.64 ***
8. Intention to Use	0.13 *	0.14 *	0.36 ***	0.50 ***	0.55 ***	0.53 ***	0.65 ***	–
*Mean_Cold Robot_*	3.39	1.98	4.45	3.69	5.50	5.83	5.76	5.04
*Standard Deviation_Cold Robot_*	0.83	0.77	1.22	1.45	1.32	1.08	1.16	1.70
*Skewness_Cold Robot_*	−0.03	0.88	−0.72	0.20	−0.63	−1.03	−1.04	−0.84
*Kurtosis_Cold Robot_*	−0.43	0.32	−0.50	−0.70	−0.37	0.72	0.48	−0.27
*Mean_Empathic Robot_*	3.39	1.97	5.20	4.33	6.07	5.78	5.79	4.95
*Standard Deviation_Empathic Robot_*	0.80	0.69	0.86	1.40	1.12	1.06	1.25	1.85
*Skewness_Empathic Robot_*	0.05	0.83	−1.45	−0.29	−1.30	−0.77	−1.13	−0.74
*Kurtosis_Empathic Robot_*	−0.49	0.52	2.35	−0.67	1.09	0.12	0.51	−0.61

Note. The values below the diagonal are taken from participants in the cold robot condition, whereas the values above the diagonal are taken from participants in the empathic robot condition. * *p* < 0.05; ** *p* < 0.01; *** *p* < 0.001.

**Table 3:** Comparisons of the cold robot and empathic robot conditions.

	Cold Robot Condition (*n* = 257)		Empathic Robot Condition (*n* = 270)		
	*M*	*SD*	*M*	*SD*	*t*
Narcissistic Admiration	3.39	0.83	3.39	0.80	0.10
Narcissistic Rivalry	1.98	0.77	1.97	0.69	0.18
Perceived Recognition	4.45	1.22	5.20	0.86	8.21 ***
Anthropomorphism	3.69	1.45	4.33	1.40	5.17 ***
Likability	5.50	1.32	6.07	1.12	5.37 ***
Perceived Intelligence	5.83	1.08	5.78	1.06	−0.47
Safety	5.76	1.16	5.79	1.25	0.34
Intention to Use	5.04	1.70	4.95	1.85	0.55

*** *p* < 0.001.

## Data Availability

See Section 2.8 for details regarding the OSF repository (anonymized dataset, video files, and full text prompts).

## Appendix A

### Perceived Recognition Scale (PRS): Items and Factor Loadings

The PRS comprised six items rated on a 7-point scale (1 = strongly disagree, 7 = strongly agree). Items were developed to operationalize perceived recognition as a self-relevant sense of being noticed, acknowledged, and valued during the interaction. Exploratory factor analysis (principal axis with direct oblimin rotation) supported a single-factor solution (eigenvalue = 4.32), accounting for 72.02% of the variance (α = 0.92).

**Table A1:** PRS items and factor loadings (PAF, oblimin).

Item Wording	Loading
1. The robot seemed to notice my emotional state.	0.78
2. The robot acknowledged my presence during the interaction.	0.66
3. The robot made me feel personally seen or recognized.	0.85
4. The robot seemed to value me as an individual.	0.91
5. I felt that the robot was attentive specifically to me.	0.86
6. The robot’s behavior made me feel appreciated.	0.82

## Appendix B

### Appendix B.1. Post Hoc Assessment of Common Method Variance and Discriminant Validity (CFA; AMOS)

Because both constructs were assessed within the same measurement context (i.e., post-manipulation self-reports completed immediately after viewing the stimulus), we conducted post hoc CFA-based diagnostics (AMOS 29; Arbuckle, 2024) to evaluate potential common method variance (CMV) and to further examine discriminant validity between the latent constructs. All models were estimated in IBM SPSS Amos (Version 29).

### Appendix B.2. Baseline Two-Factor Model

A two-factor measurement model was specified in which the 11 indicators loaded on their respective constructs (5 indicators for Robot Interaction Evaluation; 6 indicators for Perceived Recognition Scale), with the latent correlation freely estimated. Model fit was acceptable (CFI = 0.93, GFI = 0.90), $\chi^{2}(43)=288.4$, and the latent correlation between the two constructs was r=0.64.

### Appendix B.3. Single-Factor Model (CFA Analog of Harman’s Test)

To evaluate whether a single common factor could account for the covariance among the indicators, a one-factor model was estimated in which all 11 indicators loaded on a single latent factor. This model exhibited poor fit (CFI = 0.80, GFI = 0.73), $\chi^{2}(44)=796.6$, suggesting that a single common factor is unlikely to explain the observed pattern of covariation and providing initial evidence against severe CMV.

### Appendix B.4. Discriminant Validity: Free vs. Constrained Correlation

Discriminant validity was further evaluated via a nested model comparison in which the association between the two latent constructs was constrained to unity (i.e., r=1.00). Constraining the latent association worsened model fit, $\chi^{2}(44)=297.5$, relative to the freely estimated model, $\chi^{2}(43)=288.4$, $\Delta \chi^{2}(1)=9.10$, p=0.003. This finding supports discriminant validity (i.e., the constructs are empirically distinguishable).

### Appendix B.5. Common Latent Factor (CLF) Model

Finally, a common latent factor (CLF) model was estimated as an additional post hoc diagnostic for method effects. A method factor was specified to load on all 11 indicators in addition to their theoretical loadings, and the method factor was specified as orthogonal to the substantive constructs. The CLF model yielded a modest but statistically significant improvement in fit, $\chi^{2}(42)=281.4$, compared to the baseline two-factor model, $\Delta \chi^{2}(1)=7.00$, p=0.008. Importantly, the latent correlation between the two constructs decreased from r=0.64 to r=0.55, and the CLF loadings were smaller than the substantive factor loadings. Collectively, these results suggest some method-related shared variance, but the pattern of model comparisons is inconsistent with a single-factor explanation; the constructs remain empirically distinguishable. Nonetheless, because all indicators were collected in the same session and format, some shared-method influence cannot be fully excluded.
